# G-Quadruplexes as Potential Therapeutic Targets for Embryonal Tumors

**DOI:** 10.3390/molecules181012500

**Published:** 2013-10-10

**Authors:** Tarek Shalaby, Giulio Fiaschetti, Kazuo Nagasawa, Kazuo Shin-ya, Martin Baumgartner, Michael Grotzer

**Affiliations:** 1 Division of Oncology, University Children’s Hospital of Zurich, Zurich 8032, Switzerland; 2 Department of Biotechnology and Life Science, Faculty of Technology, Tokyo University of Agriculture and Technology, Koganei, Tokyo 184-8588, Japan; 3 National Institute of Advanced Industrial Science and Technology (AIST), Tokyo 135-0064, Japan

**Keywords:** embryonal tumors, G-quadruplexes, MYC, telomeres

## Abstract

Embryonal tumors include a heterogeneous group of highly malignant neoplasms that primarily affect infants and children and are characterized by a high rate of mortality and treatment-related morbidity, hence improved therapies are clearly needed. G-quadruplexes are special secondary structures adopted in guanine (G)-rich DNA sequences that are often present in biologically important regions, e.g. at the end of telomeres and in the regulatory regions of oncogenes such as MYC. Owing to the significant roles that both telomeres and MYC play in cancer cell biology, G-quadruplexes have been viewed as emerging therapeutic targets in oncology and as tools for novel anticancer drug design. Several compounds that target these structures have shown promising anticancer activity in tumor xenograft models and some of them have entered Phase II clinical trials. In this review we examine approaches to DNA targeted cancer therapy, summarize the recent developments of G-quadruplex ligands as anticancer drugs and speculate on the future direction of such structures as a potential novel therapeutic strategy for embryonal tumors of the nervous system.

## 1. Introduction

Embryonal tumors most commonly occur in the first few years of life and account for more than 25% of childhood malignancies [1]. They include medulloblastoma (MB), neroblastoma (NB), soft tissue sarcomas, nephroblastoma (Wilm’s tumor), bone tumors, retinoblastoma, hepatoblastoma, germ-cell tumors and various other rare subtypes. This broad group of childhood tumors differs fundamentally from adult onset cancers, both in their cell biology and their tissue environment. Embryonal tumors originate from immature tissue as a result of the aberrant proliferation of early precursor cells and their morphological appearance resembles that of tissues in the developing embryo and fetus [2,3]. Most embryonal tumors are unfavorable malignant tumors with a relatively high proportion of children dying due to current therapy resistant disease. It is therefore likely that improved treatments for these cancers will only be possible when the molecular events that are specific to the tumors are better understood.

MB and NB are malignant embryonal tumors of the central and peripheral nervous systems, respectively [4,5]. Childhood MB is a cancer of the cerebellum, while NB arises in the sympathetic nervous system showing heterogeneous biological and clinical features. Both MB and NB belong to the most challenging oncologic diseases of childhood that often show poor clinical prognosis. Despite intensive multimodal therapy, including surgery, chemotherapy and radiation, both high-risk NB and metastatic MB frequently acquire therapy resistance with fatal clinical outcomes. Moreover, many of the survivors suffer the risk of severe consequences from the intensive treatment; in particular children with MB who often experience long-term side effects mainly due to radiation therapy to the developing brain with high risks of severe morbidity even if cured of the tumor. Hence the development of novel therapeutic approaches based on identification of specific targets seems the most promising way forward to a better outcome for children with these unfavorable malignant tumors [6,7].

Anticancer agents that target DNA are some of the most effective agents in clinical use and have produced significant increases in the survival of cancer patients but, unfortunately, they are extremely toxic. Consequently, much effort has been put into finding agents that are more selective and there is considerable excitement that the identification of cancer-specific DNA targets will yield a new generation of less toxic therapeutics [8]. Secondary DNA structures, such as G-quadruplex nucleic acids, have recently emerged as a new class of molecular targets for DNA-interactive compounds. These elements are nonclassical four-stranded secondary structures arising from the folding of a single DNA strand that comprises stretches of tandem guanines. G-quadruplexes are found to be present in biologically important regions of DNA that are essential for cancer cells to proliferate indefinitely such as telomere and regulatory regions of oncogenes. Owing to the abundance of detailed information available regarding their thermodynamic stabilities [9] and their potential anticancer activities, G-quadruplexes are viewed as emerging therapeutic targets in oncology [10,11,12,13,14]. Several compounds that target these structures have shown promising anticancer activity in tumor xenograft models and some of them have entered Phase II clinical trials. This review examines approaches to DNA targeted therapy, describes recent developments of G-quadruplex ligands as anticancer drugs and discusses their potential as therapeutic targets, for embryonal tumors of the nervous system.

## 2. DNA as a Target for Anticancer Therapy

DNA has played a role as a successful molecular target for many of the drugs that have been used for decades in cancer therapeutics and there are good reasons to expect DNA will continue to be a clinically important target for many years to come [8]. Anticancer chemotherapies that target DNA are some of the most effective drugs in the clinical use and have produced significant increases in the survival of pediatric cancer patients [15]. Most of these chemotherapeutic are DNA-damaging agents that have been proven to cause relative potent destruction of tumor cells. However, the clinical potential of DNA-damaging agents is undermined by the adverse side effects and increased risk of secondary cancers that are consequences of the agents' genotoxicity [16].

DNA integrity is critical for proper cellular function and proliferation. DNA damage is detected by cell-cycle checkpoint proteins, whose activation induces cell-cycle arrest to prevent the transmission of damaged DNA during mitosis. If damaged DNA cannot be properly repaired cell death usually results [17]. The rationale for targeting DNA to treat cancer is based on the facts that rapidly proliferating tumor cells depend upon DNA integrity more than normal quiescent cells [16].

DNA-damaging compounds with anticanceractivity were shown to target DNA either directly or through inhibition of enzymes that control DNA integrity or provide building blocks for DNA. There are several established therapeutic modalities targeting DNA: (i) antimetabolites which are DNA antagonists that exert their activity by blocking nucleotide metabolism pathways, such as capecitabine, floxuridine, and gemcitabin as well as the canonical folic acid antagonists such as methotrexate; (ii) alkylation agents that cause direct DNA damage. These include compounds that directly modify DNA bases, intercalate between bases, or form cross-links in DNA, such as nitrogen mustards and their derivatives that directly alkylate DNA on purine bases, leading to stalled replication fork progression and subsequent cell death via apoptosis. Other DNA alkylators which are currently used in clinical therapeutics include cyclophosphamide, chlorambucil, melphalan, carmustine, lomustine, semustine, dacarbazine and temozolomide [8]. Temozolomide is a monoalkylation drug which methylates guanine residues in DNA. The most potent and efficacious agents, however such as chlorambucil and melphalan, were found to crosslink the two complementary strands of DNA, rather than just alkylating one strand. Intercalators such as actinomycins bind DNA and inhibit the activity of many enzymes that use DNA as a substrate; (iii) in addition to alkylating agents antitumor antibiotics such as doxorubicin, bleomycin and distamycin have made an important impact on the treatment of cancer patients; (iv) among the most widely and successfully used anticancer agents today are nonspecific DNA-damaging chemicals, including inhibitors of topoisomerases (TOPO) I and II and agents causing covalent modification of DNA such as mitomycin C, streptozotocin and platinum compounds [16]. Natural products which alkylate DNA bases such as mitomycin C and streptozotocin crosslink DNA on opposite strands of the double helix, resulting in a more potent effect against cancer cells compared to monofunctional alkylation. The discovery of the alkylating agent-like platinum agents such as cisplatin had a significant positive impact on anticancer drug research. Indeed, cisplatin therapy can cure over 90% of all testicular cancer cases and also has good efficacy in the treatment of ovarian, bladder, head and neck, and cervical cancers [17,18]. DNA remains a promising target for anticancer drug development, but DNA damage to normal cells as well is not a prerequisite for anti-tumor activity. The focus until recently was on double-stranded (ds) DNA structures that have been known for 60 years [19]. Chemotherapeutical drugs that are currently used in cancer therapy are thought to act through the unspecific recognition of highly ‘active’ ds DNA in cancer cells that is in replication at high frequency and therefore relatively exposed to recognition by DNA targeting molecules. Following DNA recognition by these compounds, the subsequent interaction involves either intercalation of the ligand’s planar aromatic rings between two adjacent DNA base pairs, or major or minor-groove binding (Table 1). However, nonspecific binding through electrostatic interactions with the negatively charged sugar-phosphate backbone frequently occurs. Consequently, this has driven interest in the targeting of unusual, non-canonical structures in DNA, in order to achieve selectivity while potentially reducing adverse side effects [20]. One DNA structure that has attracted significant attention as an anticancer target is the G-quadruplex [16]. Compared to duplex DNA, G-quadruplexes have much more compact structures that contain well-defined binding sites for small molecules. It has been proposed that the different structural morphology of G-quadruplex DNA, will provide different G-quadruplex recognition site for binding different G-quadruplex interacting ligands. Small organic molecules have been proposed to interact noncovalently with G-quadruplex through stacking on the ends of the G-tetrad core, groove binding, taking the place of one or more strands in the core, interacting with the backbone (core and loops), or interacting with the loop bases These G-quadruplex elements are often present in biologically important regions of DNA that greatly required by cancer cell to proliferate untimely such as telomere and regulatory regions of oncogenes such as MYC. Thanks to the abundance of detailed information available regarding their potential biological activities, G-quadruplexes are viewed as emerging therapeutic targets in oncology [10,11,12,13,14].

**Table 1:** Interaction patterns between drug/small molecules and double stranded DNA.

Drug	Mode of Binding			
	Covalent	Non covalent		
	Alkylating agents (irreversible and leads to complete inhibition of DNA processes and subsequent cell death)	Groove binders		Intercalators
		Minor groove binders	Major groove binders	
Cisplatin (DNA crosslinker)	√			
Doxorubicin (Stabilizes topoisomerase-II–DNA cleavable)	√			
Etoposide (Topoisomerase inhibitor)	√			
Methotrexate (Antimetabolite, a folic acid antagonist)	√			
(TFOs) Triplex-forming oligonucleotides (oligomers that bind in the major groove and form hydrogen bond with bases of the purine strand)			√	
(PNAs) peptide nucleic acids (with peptide-like backbone that invade the helix to form a triplex which results in the displacement of noncomplementary oligopyrimidine DNA strand)			√	
(Daunomycin) combilexins				√
Quinacrine				√
Ethidium bromide				√
Netropsin		√		
Distamycin		√		
DAPI		√		

## 3. G-Quadruplexes

Guanine (G) -rich DNA sequences are susceptible to form *in vitro* G-quadruplexes as a consequence of the propensity of guanines to associate with each other in a stable hydrogen-bonded arrangement, the G-quartet [21,22,23]. G-quartets are stabilized by a monovalent cation (Na^+^ or K^+^) localized in the centre of the structure. Both nuclear magnetic resonance and X-ray crystallographic structures of G-quadruplexes have been obtained at high resolution [24,25]. Variations in the molecularity, topology, strand orientation and glycosidic conformation of the G-quadruplex DNA provide a diverse array of structures [26]. A three-dimensional arrangement of three G-quartets can result in a variety of G-quadruplex structures. The four-stranded quadruplex structural types depend on the number and the orientation of the DNA strands. Indeed, intramolecular G-quadruplexes are comprised of one DNA strand whereas dimeric and tetrameric intermolecular quadruplex involve two and four DNA strands, respectively. G-quadruplex heterogeneity also depends on the orientation of the DNA strands (parallel or anti-parallel) and the guanine conformation (*syn* or *anti*) [24,27,28]. Despite a wealth of crystal and solution structures, it has proved difficult to define a comprehensive set of rules that specify the folding propensity of G-quadruplexes based on specific sequences, moreover it has been reported that the same sequence can adopt different G-quadruplex conformations [29]. 

There is extensive literature on proteins that have been identified to bind G-quadruplexes [30,31,32], including proteins that either facilitate or non-catalytically disrupt G-quadruplex formation, as well as helicases that catalytically unwind G-quadruplexes in an ATP-dependent manner and nucleases that cleave at G-quadruplex scaffolds [33]. Although G-quadruplex structures have only been observed *in vitro*, strong indirect evidence for their existence *in vivo* comes from the characterization of G-quadruplex DNA binding proteins, helicases, and nucleases. [26,34]. Moreover monoclonal antibodies have been used successfully to confirm their *in vivo*, existence [35] however, controversial reports exist [36,37].

Bioinformatics and molecular sequence analysis indicates that G-quadruplexes are over-represented in specific regions of the genome with key biological contexts. This includes DNA telomere ends and promoter regions (translation start sites) of several important oncogenes [21,33,38,39]. It has been shown that the formation of quadruplexes inhibits the telomere extension by the telomerase enzyme, which is up-regulated in cancer cells, as well as negatively regulating oncogene’s transcription [40,41] Because of its biological significance and antitumor potential, the G-quadruplex has attracted intense interest as an important target for drug design and development and there is a huge interest in design and development of small molecules to target these structures. A large number of so-called G-quadruplex ligands, displaying varying degrees of affinity and more importantly selectivity, have been reported [42,43].

RNA structures in the untranslated regions (UTRs) of mRNAs influence post-transcriptional regulation of gene expression. There is now a growing body of evidence that has established a link between deregulation of translational control and disruption of normal cell behavior in human diseases, especially cancers. While much of the research has been focused on DNA G-quadruplexes, there has recently been a rapid emergence of interest in RNA G-quadruplexes, particularly in the 5′-UTRs of mRNAs. The recent *in vitro* demonstrations that small molecule G-quadruplex binding ligands can selectively target RNA G-quadruplexes open up a new and attractive avenue in RNA-directed drug design. Clearly, part of the challenge is to better understand the mechanistic effects and selectivity *in vivo* environment, however, it is clear that the RNA G-quadruplex motif represents a structurally attractive scaffold for small molecule targeting and given the promising early insights into their functional effects, this represents an attractive and fertile area for future research [10,33].

## 4. G-Quadruplexes as a Potential Cancer Therapeutic Targets

### 4.1. Telomere Structure and Function

The concept of targeting G-quadreplexes as a therapeutic strategy was first developed for telomeric DNA and telomerase inhibition. Telomeres are specialized DNA–protein complexes that cap the ends of linear chromosomes and provide protection against gene erosion at cell divisions, chromosomal nonhomologous end-joinings and nuclease attacks [44,45,46]. Telomere DNA consists of repetitive TTAGGG double-stranded tracks that span ~10–15 Kb in length in humans and terminate with around 200-nt of a G-rich single-stranded overhang beyond the double-stranded region [47,48]. The single stranded DNA folds back and anneals with the double-stranded region to form a large telomeric loop, known as the T-loop [49]. As a consequence, a portion of the strand along the length of the overhang-invasion is displaced, forming a single-strand DNA region called a D-loop [50]. A group of telomere-associated proteins which help to stabilize the T-loop secondary structure are collectively called shelterins. These shelterin proteins comprise the telomere repeat factor 1 and factor 2 complexes (TRF1 and TRF2) that bind to double-stranded telomeric DNA and the protection of telomeres 1 protein (POT1) that binds the single-stranded 3` G-rich overhang. Three other interconnecting proteins (TIN2, TPP1, and RAP1) protect the telomere integrity by assisting in the T- and D-loop formation [51,52]. Telomere DNA in human cells shortens during each round of chromosome replication due to the end-replication problem [53,54]. In more than 85% of cancer cells, the telomere shortening is compensated by the telomerase enzyme that is especially up-regulated in cancer but not in somatic cells.

Telomerase is a cellular ribonucleoprotein enzyme that stabilizes telomere length by adding TTAGGG repeats to the telomeric ends of the chromosomes. Human telomerase is composed of two main components, human telomerase RNA (hTR) and telomerase reverse transcriptase TERT [55,56,57,58]. This enzyme utilizes its own RNA as a template to synthesize telomeric DNA. Together with telomere-binding proteins, telomerase confers stability on the chromosomes and counteracts the telomere-dependent pathways of cell mortality. Telomerase activity changes through life, going from a peak of activity during the first trimester *in utero*, where virtually all the tissues have active telomerase [59], to undetectable levels after birth in most somatic tissues with the exception of highly proliferative cells such as germ cells and stem cell compartments [60]. Beside telomerase, in some tumors, telomeres are maintained by an alternative lengthening of telomere (ALT) mechanism [61,62,63]. In this process telomeres are usually longer and more heterogeneous than in telomerase-positive cells. However, the exact mechanisms involved in telomere elongation are poorly understood.

### 4.2. Biological Significance of Telomeres and Telomerase during Development

Highly proliferative cell types such as embryonic cells require active and controlled telomere maintenance strategies in order to protect the integrity of their genomes effectively. Telomere length was found to be regulated during human and animal embryogenesis by a telomerase-dependent mechanism [64]. In germ line cells, human telomeres are balanced between shortening processes with each cell division and elongation by telomerase, but once the cell is terminally differentiated or mature, the equilibrium is shifted to gradual telomere shortening by repression of the telomerase enzyme [65,66,67,68,69,70,71]. Embryonic stem cells that are capable of self-renewal and differentiate to any cell type in the body, maintain high levels of telomerase activity and TERT expression [72,73,74,75]. In 20 week old human foetus after the embryonic period and most of the organogenesis is accomplished, telomerase is rapidly down-regulated and expressed only at lower levels in tissue-specific stem cells [76,77].

### 4.3. Telomeres and Telomerase Activity during Tumor Development

Despite the impressive advances that have been made in cell and molecular biology, how embryonal tumor cells are actually initiated and progress is still widely debated. The concept that the incidence of cancer rises exponentially in the final decades of life due to the sequential accumulation of the somatic mutations does not really fit the onset of pediatric cancers that develop and manifest early in childhood. Identification of the cells that mediate tumor initiation in childhood cancer and finding out the information that is necessary for the cell to transform into a neoplastic cell should provide an important baseline for better treatment of childhood embryonic cancers [78,79].

Different hypotheses have been postulated in the literature: one assumes that a somatic differentiated cell can dedifferentiate or reprogramme to regain properties associated with cancer cells whereas others claim that a stem cell is needed to initiate the carcinogenic process [80]. The first model scenario depends on the hypothesis that rapid proliferation of the telomerase negative dedifferentiated somatic cells can lead to shortened telomeres that may promote chromosomal and genomic instability which then primes the cell to become cancerous. In a later stage telomerase is then activated and stabilizes the previously shortened telomeres, thereby prolonging the lifespan of cancer cells. This hypothesis has been supported experimentally by the observation that almost all malignant cancers have telomerase activity, despite their shortened telomeres [65,81,82,83,84]. Indirect support for this view comes from the observations that benign or pre-cancerous lesions are telomerase silent [81]. Moreover high telomerase levels are found to correlate with worse clinical outcomes [85]. This model implies that telomerase activation in cancer is an induced or aberrant function in otherwise enzyme-deficient somatic cells destined for senescence [86]. The second interesting hypothesis is that the tumor cells are telomerase positive not because of TERT expression under a selective pressure, but because they are derived from the oncogenic transformation of a stem cell or a pluripotent early precursor cell which has retained its telomerase activity [76,86,87]. This concept has been proposed for several tumors [88] and supported by a number of reports demonstrating the presence of cancer stem cells in different adult cancers [89,90]. In pediatric malignancies the cancer stem cell hypothesis was recently described in studies performed on leukaemia, where it was shown that a single cell with stem cell markers had the capability to induce the disease in mice [91]. More recently, cancer stem cells have also been isolated from solid embryonal tumors such as MB, NB, Ewing’s sarcoma, RMS and HB [92,93,94,95,96,97]. The second model scenario highlights the importance of telomere length maintenance in stem cell populations to facilitate cell division that is required for tissue homeostasis. However there has to be a balance between maintaining regenerative potential, on one hand and tumor suppression on the other. One mechanism that may contribute to adjust this balance is the length of telomeres *per se*, whereby stem cells may need to maintain telomeres at a length that provides sufficient replicative capacity for tissue homeostasis, versus the requirement to minimize telomere length and replicative capacity as a tumor suppressive mechanism [98]. There is a suggestion that during the tumorigenesis process telomere erosion may have evolved to a level where telomeric repeat sequences are too short to provide a functional substrate for telomerase enzyme activity [99]. In this scenario, as telomeres shorten with each cell cycle the “sticky” ends of chromosomes become prone to fusions [100] leading to subsequent chromosomal instability [100,101,102,103] and offering a mechanism for a continuous rearrangement of chromosome structure that might contribute to oncogene amplification and tumor suppressor gene deletion [104,105]. In fact, concurrent telomere shortening and genomic instability have been observed in the majority of embryonic tumors including: Wilms’ tumor [106,107], MB [108,109], NB [110,111] and rhabdomyosarcoma [112,113]. The view represented by the stem cell origin of embryonal tumors implies that the genetic alterations which lead to cancer accumulate in embryonal stem cells rather than mature cells. However, an alternative opinion held is that it is important to separate tumor-initiation and tumor-propagation; this may not involve the same cell type as the tumor-propagating cell may be a much differentiated progeny of the tumor-initiating cell. Hence improved therapeutic efficacy may be achieved by targeting both cell types which drives malignant progression as well as thesewhich initiates and maintains the stem cell pool of the tumor [114].

Ultimately whether embryonal cancer cells reactivate the telomerase or up-regulated telomerase activity, the telomere maintenance process seems to play a crucial role in the initiation and progression of cancer. Since telomerase is not expressed in most normal human cells, this has led to the development of targeted telomerase cancer therapeutic approaches which are at present in advanced clinical trials.

### 4.4. Significance of Telomere Biology in Embryonal Tumors of the Nervous System

The erratic clinical behaviour of pediatric embryonal tumors suggests a variable proliferative potential, thus making them attractive candidates for the study of telomere maintenance biology as a possible prognostic marker and/or therapeutic target. Elevated telomerase activity and telomere shortening could be signs of the excessive cell divisions experienced by cancer cells and could reflect the stage of malignancy and disease prognosis [115]. Down-regulation of telomerase activity has been shown to induce cancer cell growth arrest and differentiation, which might predict a close correlation between telomerase activity levels and clinical outcome, while tumors with sustained telomerase activity might therefore become choice targets for telomerase directed therapy [116,117,118,119]. Telomere maintenance biology have been studied in the majority of embryonic tumors including Wilms’ tumor [106,107,120], Ewing’s sarcoma [121,122,123], hepatoblastoma [8,124], MB [108,109,125,126], NB [127,128] and rhabdomyosarcoma [112,129]. We will focus below on pediatric malignancies of the central and peripheral nervous system MB and NB.

#### 4.4.1. Neuroblastoma

NB is the most common extra-cranial solid tumour of childhood and accounts for at least 15% of cancer-related deaths in children [4]. NB is derived from primitive cells of the sympathetic nervous system and so it can be found anywhere along the paravertebral sympathetic chain or in the adrenal gland [130,131]. The clinical outcome of NB can range from complete regression (mainly in infants) to rapid tumor progression and metastasis with poor prognosis [132]. Identification of the most common genomic alterations associated with the disease has allowed the classification of NB into low-, intermediate- and high-risk groups [4,133]. Unfavorable tumors are characterized by deletions of 1p or 11q, unbalanced gain of 17q and/or amplification of *MYCN* [134].

Studies by several independent laboratories aimed at understanding the dynamics of telomere-telomerase interaction in NB suggested that telomerase activity is a robust prognostic indicators [127,128,135,136,137] and can discriminate between prognostically different subsets of NB [85,138,139]. Hiyama *et al.* reported that telomerase is expressed in 94% of NB patients’ samples, but not in benign ganglioneuromas or adjacent adrenal tissues: 75% of tumors with high telomerase activity had a poor prognosis, 97% of tumors with low telomerase activity had a good prognosis and 100% of tumors with no detectable telomerase activity regressed [85,135]. Similarly, in a study of a large cohort, telomerase activity was detected in 39/133 (29%) tumors including 25/41 (61%) Stage 4, 8/23 (35%) Stage 3, 0/13 (0%) Stage 2, 2/32 (6%) Stage 1 and 4/24 (17%) Stage 4S NB. In this study telomerase activity emerged as an independent predictor of clinical outcome with greater prognostic impact than the MYCN status and even the clinical stage [140]. The level of RNA subunit of telomerase (hTR) has also been found to be associated with the clinical stage of NB at diagnosis [141]. High expression of hTR was associated with advanced disease and with unfavorable prognosis, while most patients with weak or absent hTR expression were found to belong to early tumor stages [138,141]. NB patients classified as 4S stage, known to have a good prognosis and usually demonstrating spontaneous regression, were found to exhibit short telomeres and to express no detectable telomerase activity at diagnosis, in contrast to patients with progressive disease [135,136]. Hence it has been hypothesized that the aggressive tumors express telomerase (and therefore have stabilized telomeres), whereas the regressing tumors may have absent or low levels of telomerase activity (allowing telomeres to continue shortening). In a retrospective study on 124 NB, Krams *et al.* have shown that both spliced and full-length hTERT transcripts were significantly associated with *MYCN* amplification while full-length hTERT transcripts were highly predictive of poor outcome [142]. In a recent work we examined telomestatin, a G-quadruplex interactive agent, for its ability to inhibit telomere maintenance of neuroblastoma cells. In this study treatment with telomestatin resulted in telomerase inhibition, telomere shortening, cell growth suppression and induction of apoptosis through disruption of telomere maintenance [143].

#### 4.4.2. Medulloblastoma

MB is a malignant, invasive tumor of the cerebellum and the most common primary pediatric malignancy of the central nervous system. Classified as a primitive neural ectoderm tumor that is thought to arise from granule cell precursors. The standard of care consists of surgery, chemotherapy and age-dependent radiation therapy. Despite aggressive therapy approximately 30% of MB patients remain incurable. Moreover, for long-term survivors, the treatment related sequelae are often debilitating. Side effects include cerebellar mutism, sterility, neurocognitive deficits and a substantial risk of developing secondary cancers. Hence more effective and targeted therapies are certainly needed [144].

In contrast to NB, data on the role of telomere/telomerase biology in MB are scarce and examination of the few reports that do exist yields conflicting results. Studies have shown that large increases in chromosomal material in the 5p15 region, where the TERT gene is located, are detectable in MB, suggesting that the TERT gene could be amplified in CNS embryonal tumors [83,145,146]. Fan et al. used differential PCR and real-time RT-PCR to determine the relationship between TERT gene copy number, TERT mRNA expression and clinical outcome in CNS embryonal tumors including MB [147]. The group found that the TERT gene was amplified in 42% of 36 primary MB samples examined. The TERT amplification was found to correlate with the increased expression of TERT mRNA in almost all the tumors, while MB patients with increased TERT expression in their tumors showed a trend towards worse clinical outcomes. The authors suggested that changes may have happened at the TERT locus during the evolution of MB, indicating a possible role for telomerase in the pathogenesis of MB [147]. Other groups, including our laboratory [148,149,150,151], detected telomerase enzyme activity in cultured MB cells *in vitro*. Our lab investigated the mRNA expression level of TERT in 50 primary MB samples and compared it with seven normal brain samples. 76% of the primary MB samples had upregulated TERT mRNA expression [148,149,150,151]. While a positive correlation between TERT mRNA expression and telomerase activity was detected in MB cell lines, no correlation was found between telomerase activity and telomere length. Treatment of MB cell lines with the telomerase inhibitor epigallocatechin gallate displayed strong dose dependent proliferation inhibitory effects against telomere repeat amplification protocol (TRAP)-positive MB cell lines [148,149,150,151]. Our results suggest that inhibition of telomerase function could represent a novel experimental therapeutic strategy in childhood MB. In contrast, however, by screening a heterogeneous group of brain tumors for telomerase activity, MB was found to be the only telomerase negative in the series of brain tumors tested [152,153]. Hence these results may provide a stimulus for future research aimed at uncovering the real role, if any, that telomere maintenance might play in the pathogenesis of MB.

### 4.5. Significance of the myc Oncogene Family in Embryonal Tumors of the Nervous System

#### 4.5.1. *MYC* in neuroblastoma

The *myc* family of nuclear oncogenes contains three well-characterized members, c-myc (MYC), MYCN, and L-myc. These genes encode related but distinct nuclear proteins that can contribute to tumorigenic conversion both *in vitro* and *in vivo*. However, each gene displays a unique activation pattern that partially reflects the distinctive expression of each gene during normal tissue as well as during tumor development [153]. *MYCN* amplification in NB has been established as a predictive marker for poor outcome which is associated with a survival rate of 15%–35% [154,155]. MYCN is vital for proliferation, migration and stem cell homeostasis while decreased levels are associated with terminal neuronal differentiation. In addition, high risk tumors without *MYCN* amplification frequently express elevated levels of MYC [156]*.* Recent microarray data from NB patients showed that genes in the MYC pathway significantly correlated to poor survival independent of *MYCN* amplification [157]. By using these expression profiles, the authors identified patients with adverse outcomes that initially were diagnosed as low or intermediate risk [157], emphasizing the importance of MYC signaling in NB biology. Retinoic acid has been shown to downregulate MYCN expression and to induce neuronal differentiation of NB cells *in vitro*. Together, these findings indicate that MYC signaling is important in maintaining an undifferentiated phenotype and that inhibition of MYC could contribute to less aggressive tumors and maybe even lead to new and improved therapies for high-risk patients (reviewed in [158]). Several labs have explored the effect of down regulating MYCN expression in *MYCN*-amplified NB cell lines using antisense or RNA interference approaches *in vitro* and *in vivo* [159,160,161,162,163,164,165]. Collectively these studies demonstrated that decreased MYCN expression in NB cells leads to growth arrest, apoptosis and/or differentiation. The results of these studies indicate that MYCN could be a promising therapeutic target for NB. However the use of antisense or RNA interference as a therapeutic strategy in the clinic has been limited due to insufficient delivery and specificity problems. It is therefore of particular relevance to find an alternative approach to achieve a better targeting of MYCN in NB in view of discovering new therapeutic targets. 

#### 4.5.2. *MYC* in medulloblastoma

MYC has emerged as an important modulator and prognostic indicator of MB malignancy [166,167,168]. Amplification of *MYC* has been reported in 5%–15% of MB overall, while amplification of *MYCN* has been found in ~10% of cases [169,170,171]. MYC expression and amplification have been associated with poor patient prognosis [166,172] and with the prognostically dismal large cell/anaplastic MB subtype [167,171,173]. MYCN was shown to be upregulated by Hh signaling and to mediate the effects of Hh activation on the proliferation of cerebellar granule precursors [174,175]. MYC was found to cooperate with Hh by enhancing tumorigenicity of nestin-expressing neural progenitors that are present in the cerebellum at birth and that can act as the cells-of-origin for MB [176] and reviewed in [177]. Based on the above, MYC appears to play a central role in deviating many of the signaling pathways to specific effectors involved in MB pathogenesis, therefore it represents an attractive target for the therapy of these neoplasms. However, contrary to the substantial amount of preclinical studies in NB, the investigation of MYC-specific therapeutic approaches in MB is still in its infancy. Unfortunately, clinically useful inhibitors of MYC are not available to date and efforts to develop such drugs would certainly be needed. Our lab as well as others used an antisense approach to silence MYC [178], which led to an inhibition of cell proliferation and to an arrest of the cell cycle in the S phase. The synthetic quassinoid derivative NBT-272, is currently under investigation in our group, on the basis of previous findings obtained in a panel of MB-derived cell lines [179]. In this report, NBT-272 was able to reduce cell proliferation and to block cell cycle progression [180]. We evaluated recently the effects of G-quadruplex targeting compound S2T1-6OTD on a representative panel of human MB and atypical teratoid/rhabdoid AT/RT childhood brain cancer cell lines. S2T1-6OTD is a novel telomestatin derivative that is synthesized to target G-quadruplex-forming DNA sequences in the MYC promoter. We showed that treatment with S2T1-6OTD reduced the mRNA and protein expressions of MYC and hTERT, which is transcriptionally regulated by MYC, and decreased the activities of both genes. In remarkable contrast to control cells, short-term (72-hour) treatment with S2T1-6OTD resulted in a dose and time-dependent antiproliferative effect in all MB and AT/RT brain tumor cell lines tested with IC50 at micromolar level. Under conditions where inhibition of both proliferation and MYC activity was observed, S2T1-6OTD treatment decreased protein expression of the cell cycle activator cyclin-dependent kinase 2 and induced cell cycle arrest. Long-term treatment (5 weeks) with nontoxic concentrations of S2T1-6OTD resulted in a time-dependent (mainly MYC-dependent) telomere shortening. However, telomestatin is known to bind to the G-quadruplex in the *TERT* promoter, and this may mediate at least part of its effect on TERT [180]. Telomere shortening was accompanied by cell growth arrest and followed by cell senescence and induction of apoptosis in all five cell lines investigated [181,182]. Ref.181 missing *In vivo* animal testing will now be needed to determine whether S2T1-6OTD may represent a novel therapeutic strategy for childhood brain tumors.

## 5. Targeting G-Quadruplex as a Novel Anticancer Strategy

### 5.1. Targeting Telomere Maintenance

The interest in telomere maintenance mechanisms in a cancer therapeutics context came to light following the observations that immortality of human cancer cells is intimately related to the maintenance of the ends of human chromosomes [183,184]; in addition over 85% of human tumor samples including cancer stem cells are telomerase-positive [56,57,58]. In fact no other tumor-associated gene is as widely expressed in cancer. This concept was coupled with the remarkable reports by Hahn and colleagues’ showing that cloning a mutant TERT gene into a cancer cell causes the cell to lose the ability to form tumors in mice, leads to shortening of telomeres and forces the cell into replicative senescence [183,185]. Soon afterwards cumulative reports continued to demonstrate and provide evidence for the genetic validation of telomere maintenance as an anticancer target [186,187,188,189].

**Figure 1:**
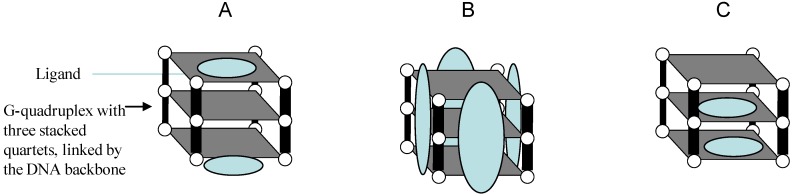
Interaction modes between G-Quadruplex structures and ligands.

Telomere DNA in human cells shortens during each round of chromosome replication due to the end-replication problem [53,54]. In most cancer cells the telomere shortening is compensated by the telomerase enzyme. Optimal telomerase activity requires the unfolding of the single-stranded 3' ends of telomeric DNA substrate that gives access to the telomerase RNA to allow priming and elongation of the telomere length. To this end, telomerase hybridize to the single-stranded 3′ ends of telomeric DNA and add new nucleotides in order to maintain telomere length and accordingly uphold the proliferative ability of the cancer cells [81,190]. This link to cancer biology propelled the development of new strategies to limit cancer cell growth using the interference with telomere maintenance via telomerase inhibition [191,192]. One recent approach to telomerase inhibition involves the sequestration of the single-stranded 3′ ends of telomeric DNA into higher-order quadruplex structures [190,193]. A desired ligand would recognize a G-quadruplex structure formed by human telomeric sequences with high affinity and specificity. Many of the reported G-quadruplex ligands contain planar aromatic rings, which can interact with human telomeric G-quadruplex by stacking on the terminal G-tetrads (Figure 1).

Small organic molecules have been proposed to interact noncovalently with G-quadruplex through (**A**) tetrad end stacking, (**B**) groove binding and (**C**) intercalation between the two G-tetrads.

In addition to the end-stacking binding mode of the aromatic rings, some ligands also contain other moieties that can recognize loops by stacking with loop bases or forming intermolecular hydrogen bonds or recognize the backbone with electrostatic interactions. The grooves in G-quadruplexes can also be recognized through hydrogen bonds or hydrophobic interactions Alternatively the G-rich human telomeric DNA strand can be trapped in a G-quadruplex structure with a linear guanine-containing molecule based on a different backbone, such as PNA [194].

The formation of a quadruplex-ligand complex at telomere ends appears to be equivalent to the exposure of damaged DNA, since it elicits a rapid DNA damage response that is lethal to the affected cancer cells [195]. Several selective drug-like small molecule ligands were developed to target the quadruplex forming 3′-telomeric end DNA sequences and a growing number of different cancer research groups including our lab, started to use some of these compounds to test cancer growth inhibitory effects [196,197,198]. Most of the small molecules discovered showed a strong ability to stabilize these motifs and were successful in telomerase inhibition, thereby identifying the human telomeric DNA G-quadruplexes as attractive potential targets for cancer therapeutic intervention [8,187]. However lack of selectivity towards telomere quadruplex motifs has been also reported [187].

Interestingly some G-quadruplex-targeting compounds have been shown to disrupt telomere capping and induce rapid apoptosis in cancer cells, even in the absence of telomere shortening [186,187]. This finding cannot be explained solely by telomerase inhibition. Rather, it indicates to a certain extent that the direct target of these ligands is telomere dysfunction rather than telomerase inhibition. Furthermore, G-quadruplex-targeting compounds have also been shown to inhibit the alternative lengthening of telomeres (ALT) pathway, which maintains telomere stability in a telomerase-independent manner in around 15% of cancer cells whereby telomerase is not activated [199].

### 5.2. Targeting G-quadruplexes in Oncogene Promoters

In addition to their existence in telomere sequence, bioinformatic analyses combined with biophysical and structural investigations have highlighted the relative abundance of putative G-quadruplex forming sequences in promoter regions of oncogenes close to their transcription start sites [200,201]. Oncogenes with putative G-quadruplex forming sequences in their promoters includes: c-kit [202,203]; k-ras [204]; hTERT [205,206]; Bcl-2 [207]; VEGF [208]; HIF-1[209]; c-myb [210]; c-myc [211,212,213] PDGF-A [214] pRb [215] reviewed in [216]. Interestingly it has been shown that the potential for quadruplex formation is higher within oncogenes` promoters compared to tumor suppressor genes [199,217]. Considerable focus has been placed on the *MYC* gene promoter making it the most extensively studied system for the G-quadruplex formation [112,199,211,218,219] important regulator of a wide array of cellular processes necessary for normal cell growth and differentiation and its dysregulation is one of the hallmarks of many cancers [220]. Hence studying MYC transcriptional activation is critical for understanding developmental and cancer biology, as well as for the development of new anticancer drugs.

Following the successful demonstration that the activity of telomerase can be inhibited by small molecule-induced stabilization of telomeric G-quadruplex, Hurley and co-workers reported the seminal discovery of a potential G quadruplex structure in the nuclease hypersensitive element III1 (NHEIII1) of the promoter region of the MYC oncogene that controls up to 80%–90% of the transcriptional activity of this gene [221,222]. The authors further demonstrated that the transcriptional repression of MYC can be achieved by induction of putative G-quadruplex formation by a small molecule [223]. Evidently, MYC transcription was inhibited by the putative formation of the G-quadruplex structure in the promoter region, thus suppressing oncogenic expression [212]. Additional support for this idea came from cellular experiments in both our and other labs, whereby transcription of MYC and hTERT was inhibited upon addition of the G-quadruplex-interactive compound TMPyP4 [36] or the telomestatin derivative S2T1-6OTD [181,182]. Unlike telomeric G-quadruplexes, which can be formed from the single-stranded DNA template at the 3′ end of human telomeres, G-quadruplexes in gene promoter regions are constrained by the duplex nature of genomic DNA. It has been found that each of the single-stranded elements, in gene promoter double stranded DNA, could be a precursor to the formation of secondary DNA structures (G-quadruplexes and i-motifs on the G-rich and C-rich strands, respectively) reviewed in [224]. Each of the single strands have the ability to form isomorphic protruding structures, which are in equilibrium with the double-stranded B-DNA form of that region [181,222]. The protruding G-quadruplex structure and the I-motif formed on the opposite strand keep the two DNA strands separated and prevent the formation of the basal transcriptional complex. When this promoter region is in B-DNA form the transcription can be initiated [225]. Compounds that bind to and stabilize the G-quadruplex conformation have been shown to reduce MYC expression and are antitumorigenic, supporting the proposed hypothesis [182,226,227].

## 6. G-Quadruplex-Interactive Small-Molecules

The therapeutic potential of G-quadruplexes has resulted in a rapidly increasing number of studies in which small-molecule ligands have been used to act as G-quadruplex stabilizers. Several hundreds of small molecules that interact with G-quadruplexes have now been described in the literature [226,227,228,229,230], however cellular and *in vivo* data are only available for a small number of these compounds [193]. These compounds may be of natural origin such as cryptolepine, berberine and telomestatin or synthetic ones such as BSU1051, RHPS4, TMPyP4, pyridine or phenanthroline dicarboxamides, triazines, PIPER or bi- and trisubstituted acridines such as BRACO19. Other potential G-quadruplex-targeting drugs including quindoline derivatives and 307A has shown various levels of selectivity and potency in binding to G-quadruplexes (Table 2). Many such agents are currently in various stages of preclinical testing and some of them will likely enter the clinic in the near future [199].

**Table 2:** Small molecules showed antitumor activity in both adults and pediatric cancers. Reviewed in [229,230].

Ligand	Tumor model tested	Antitumor activity	Reference
Telomestatin	Neuroblastoma, myeloma, acute leukemia and glioma stem cells	Telomerase inhibition, telomere length reduction Inhibition of proto-oncogene c-Myb expression Antiprolifrative activity, apoptosis induction and increased chemosensitivity Impairs cancer stem cell survival and growth	[231,232,233,234,235,236,237,238,239,240]
S2T1-6OTD (telomestatin synthetic Derivative)	Paediatric brain cancer (Medulloblastoma and atypical teratoid/rhabdoid)	MYC and hTERT inhibition Telomere shortening Cell cycle arrest and tumor cell’s growth inhibition	[182]
HXDV (telomestatin syntheticDerivative)	A panel of normal/cancer telomerase- and ALT-positive cell lines	Inhibition of cell growth independently of telomerase activity M-phase cell cycle arrest Mitotic defects Induction of apoptosis	[241]
TMPyP4 (Cationic porphyrin)	Myeloma, cervical, pancreatic, breast, colon, prostate cancer and osteosarcoma, neuroblastoma and retinoblastoma	MYC and hTERT inhibition Blockage of telomerase elongation Antiproliferative activity	[242,243,244,245,246]
SYUIQ-5 and other quindoline derivatives	Leukemia, Burkitt’s lymphoma, human epithelial carcinoma, nasopharyngeal carcinoma	MYC and hTERT inhibition Antiproleferative activity cellular senescence; apoptosis induction	[2,41,58,226,247,248]
Tetrasubstituted napthalene diimides ligands	Brest, prostate cancer , and lung adenocarcinoma	Inhibition of telomerase, activity	[249]
Triazine derivatives	Melanoma, mouth, lung, colon cancer as well as, lung adenocarcinoma	Impairs the splicing machinery of hTERT by stabilizing quadruplexes located in the hTERT intron 6 Telomere shortening Antitelomerase activity, senescence and cancer cell growth arrest	[250,251,252,253,254]
Trisubstituted acridine (AS1410)	Breast and lung cancer	Synergistic activity in combination with cisplatin	[255]
BRACO-19 3,6,9-trisubstituted acridine	Breast and prostate cancer, uterus and vulval carcinoma	Decreases hTERT expression Induction of cellular senescence; cessation of cell growth	[252,253,256,257,258,259,260]
Pentacyclic acridines (RHPS4)	Melanoma, breast and vulval cancer	Telomerase inhibition Telomere capping disruption Apoptosis via PARP-1 activation Cell cycle perturbations and decrease in cancer cell growth Increased sensitivity to chemotherapy	[253,259,261,262,263,264,265,266]
4,5-di-substituted acridone	Breast and lung cancer	Inhibition of telomerase activity Telomere length shorting Senescence induction, cancer cell growth inhibition	[267]
Anthracene derivatives	Melanoma, colon cancer and osteogenic sarcoma	Telomere dysfunction Senescence and cell growth impairment	[268]
Amidoanthraquinone derivatives	60 different human cancer cell lines	Telomerase inhibition High anti-proliferative activity	[269]
Perylene derivatives	Melanoma, colon and breast carcinomas and osteosarcoma and colorectal carcinoma cell	Selectiv for telomeric G-quadruplex with respect to duplex genomic DNA. Telomerase inhibition.	[270,271]
Macrocyclic pyridyl polyoxazoles	Oral carcinoma and breast cancer	Selective for G-quadruplex DNA with no stabilization of duplex DNA or RNA Cytotoxic to cancer cell line	[272,273]
Triethylene tetramine (TETA)	Brest cancer and human epithelial carcinoma	Telomerase activity inhibition Induction of cellular senescence	[274,275]
Bisquinolinium pyridine dicarboxamide compound (360A)	Cervical cancer and colorectal carcinoma	Telomere aberrations Impair mitotic cell progression and lead to cell death.	[276,277]
307A 2,6-pyridin-dicarboxamide derivative	Glioma and osteosarcoma	Equipotent against MYC and telomeric G-quadruplex-forming sequences Inhibiting proliferation and induce apoptosis	[278]
Bisantrene derivatives (An1,5)	Melanoma and osteogenic sarcoma	Inhibit telomerase activity long-term cell growth inhibition in both telomerase- and ALT-positive cancer cell lines Induction of senescence and autophagy	[268]

Two of the most studied small molecules are telomestatin for telomeric and TMPyP4 for MYC quadruplexes. Telomestatin is one of the most potent and selective G-quadruplex binding small molecules known so far. Telomestatin is a natural product isolated from *Streptomyces anulatus* 3533-SV4 that acts by inhibiting the telomerase activity of cancer cells [232]. It induces the formation of basket-type G-quadruplex structures in the telomeric region, impairs telomere replication and inhibits growth of tumor cells [233]. There is compelling evidence that telomestatin as well as the synthetic BRACO19 and RHPS4 act not only by inhibiting the catalytic function of telomerase, but also by uncapping telomerase from the 3′ ends of telomeres, as reviewed in [234]. Evidence of antitumor activity in various xenograft models has been reported for telomestatin, adding to its widely displayed anticancer activity in human cancer cells including multiple myeloma, acute leukemia, NB, cells where it inhibited telomerase activity, reduced telomere length and caused apoptotic cell death [234,235,236] and also increased chemosensitivity in some of these malignancies. Recently telomestatin was found to impair glioma stem cell survival and growth through the disruption of telomeric G-quadruplex and inhibition of the proto-oncogene, c-Myb [237]. However no study of telomestatin has as yet progressed to clinical evaluation.

TMPyP4 (mesotetra (N-methyl-4-pyridyl) porphine) TMPyP4 is a G-quadruplex-targeting ligand that has been used in a large number of studies. TMPyP4 is known to bind strongly to DNA quadruplexes by stacking on the G-tetrads at the core of the quadruplex, resulting in telomerase inhibition [238]. *In vitro* and *vivo* data showed that, TMPyP4 displayed an antiproliferative effect on cancer cells [239] through its interaction with the G-quadruplex formed in the promoter region of *MYC* gene [223] that consequently downregulate MYC and its downstream targets [36]. However, a major hurdle in the development of TMPyP4 as a G-quadruplex target agent is its ability to bind to duplex DNA [279] and triplex DNA [280]. Thus, attempts have been made to generate second-generation cationic porphyrins with high selectivity for G-quadruplexes [281]. A more selective agent for the MYC G-quadruplex is the telomestatin derivative S2T1-6OTD, which has been shown to reduce the expression of MY*C* and TERT in childhood MB and in AT/RT tumor cells and has potent antiproliferative effects [181,182].

### G-Quadruplex-Targeting Drugs in Clinical Trials

Quarfloxin (also known as CX-3543 or itarnafloxin), is a first-in-class G-quadruplex-interacting compound that has reached Phase II clinical trials for the treatment of neuroendocrine/carcinoid tumors. Quarfloxin is a fluoroquinolone-based antitumor agent derived from norfloxin via A-62176 and QQ58. The latter compound has a mixed mechanism of action as a topoisomerase II poison and a G-quadruplex interactive compound [224,279,280,281,282]. It was shown by the Hurley laboratory, the developer of this small molecule, that quarfloxin is highly selective for the G-quadruplex versus duplex or single-stranded DNA, and it is more selective for the MYC G-quadruplex versus other G-quadruplexes [282]. Quarfloxin disrupts the interaction between the nucleolin protein and a G-quadruplex DNA structure in the ribosomal DNA (rDNA) template, a critical interaction for rRNA biogenesis that is overexpressed in cancer cells. Disruption of this interaction may result in the inhibition of ribosome synthesis and tumor cell apoptosis [283,284]. Owing to its potent *in vivo* efficacy in a broad range of tumors, quarfloxin is currently in Phase II clinical trials as a single-agent therapy for neuroendocrine tumors.

In addition to being potential drug targets, DNA G-quadruplexes have also been shown to be potential cancer therapeutics themselves. AS1411 (Antisoma, London, UK) is a guanosine-rich 26-base G-quadruplex-forming oligonucleotide aptamer that can inhibit the growth of malignant cells by inducing apoptosis. AS1411 is currently in Phase II trials for the treatment of renal cancer and acute myeloid leukemia. AS1411 has been shown to have cancer-selective antiproliferative activity against a wide range of malignant cell types [199,285]. Finally, small molecules are not the only way to target nucleic acid structures, a high-affinity single-chain antibody has been developed which is highly specific for antiparallel telomeric repeats from *Stylonychia lemnae* macronuclei and binds to the telomeric repeats *in vivo*, not only demonstrating the concept of antibodies as ligands, but also providing one of the first key pieces of evidence that these structures are present *in vivo*, while the design and synthesis of new high affinity G quadruplex ligands will provide new drug candidates and molecular probes [35,286].

## 7. Conclusions

Both MB and NB belong to the most challenging oncologic diseases of childhood that often show poor clinical prognosis. Despite intensive multimodal therapy high-risk NB and metastatic MB frequently acquire therapy resistance with fatal clinical outcomes, hence the development of novel therapeutic approaches based on identification of specific targets seems the most promising way forward to a better outcome. There is good evidence to suggest that MYC oncogene expression and the telomere maintenance process in pediatric embryonal tumor cell populations are important in facilitating cell divisions required for cancer cell immortalized proliferation, thus making both of them attractive candidates for possible therapeutic targets. The discovery of G-quadruplex structures in specific, biologically important regions of the genome that are greatly required by cancer cells to proliferate, made them a significant drug target and ever since several compounds targeting these structures have been discovered and shown promising anticanceractivity. The therapeutic advantages of such a novel approach to anticancer drugs resides in the evidence that G-quadruplex ligands selectively impair the growth of cancer cells without affecting the viability of normal cells [240,244,249], together with the ability of some of these compounds to exert an antitumor activity in different *in vivo* models and to induce antiproliferative effects also in ALT cells [61,62,63]. However the compounds discovered so far are moving very slowly to the clinical setting and most of them have not yet progressed past pre-clinical investigation [287]. To advance further more efforts should be directed toward better understanding of the biological functions of G-quadruplexes *in vivo*, together with additional progress on the development of small molecules with realistic drug-like structures, higher selectivity and decreased side effects. Although research activity on telomeric and oncogeneic quadruplexes in embryonal tumors is still in its infancy, it is hoped that their therapeutic potential will encourage more future research in this exciting new area of molecular targeted therapy for pediatric oncology aimed towards a successful strategy for curing childhood cancer. The promise and potential is high: the challenges are considerable but surmountable.
